# Contraceptive Method Use Among a Population-Based Cohort During the South Carolina Choose Well Initiative

**DOI:** 10.1001/jamanetworkopen.2024.8262

**Published:** 2024-04-24

**Authors:** Nathan Hale, Jusung Lee, Jordan de Jong, Michael G. Smith, Amal J. Khoury

**Affiliations:** 1 Center for Applied Research and Evaluation in Women’s Health and Department of Health Services Management and Policy, College of Public Health, East Tennessee State University, Johnson City; 2 College for Health, Community and Policy, University of Texas at San Antonio, San Antonio

## Abstract

**Importance:**

Evaluating the impact of statewide contraceptive access initiatives is necessary for informing health policy and practice.

**Objective:**

To examine changes in contraceptive method use among a cohort of women of reproductive age in South Carolina during the Choose Well contraceptive access initiative.

**Design, Setting, and Participants:**

In this cohort study, baseline data from the initial Statewide Survey of Women administered from October 1, 2017, to April 30, 2018, to a probability-based sample of women of reproductive age in South Carolina and a peer state (Alabama) were linked with 3 follow-up surveys given in 2019, 2020, and 2021. Responses about contraception use from the initial survey were compared with responses across follow-up surveys using the regression-based differences-in-differences method. Data analysis was performed from October 2023 to February 2024.

**Exposure:**

The South Carolina Choose Well contraceptive access initiative seeks to fill contraceptive access gaps and increase provision of a full range of contraceptive methods through engagement with a wide range of health care organizations across the state.

**Main Outcomes and Measures:**

Changes in contraceptive method use, including long-acting reversible contraception (LARC), intrauterine devices (IUDs), implants, short-acting hormonal injection, and barrier or other methods between the baseline survey (2017-2018) and 3 subsequent surveys (2019-2021).

**Results:**

A total of 1344 female participants (mean [SD] age, 34 [7] years) completed the first survey (667 in Alabama and 677 in South Carolina). Use of LARC significantly increased in South Carolina (119 [17.6%] to 138 [21.1%]) compared with Alabama (120 [18.0%] to 116 [18.1%]; *P* = .004). Use of IUDs increased in South Carolina (95 [14.0%] to 114 [17.4%]) compared with Alabama (92 [13.8%] to 102 [15.9%]; *P* = .003). These associations persisted in the adjusted analysis, with a significant increase in the odds of LARC (adjusted odds ratio, 1.24; 95% CI, 1.06-1.44) and IUD (adjusted odds ratio, 1.19; 95% CI, 1.06-1.32) use at follow-up in South Carolina compared with Alabama.

**Conclusions and Relevance:**

In this cohort study of 1344 participants, increases in the use of IUDs in South Carolina were noted after the implementation of the South Carolina Choose Well initiative that were not observed in a peer state with no intervention. Our findings may provide support in favor of statewide contraceptive access initiatives and their role in promoting access to reproductive health services.

## Key Points

**Question:**

To what extent has contraceptive method use changed during a statewide contraceptive access initiative in South Carolina compared with a peer state with no intervention?

**Findings:**

In a cohort study of 1344 women of reproductive age from South Carolina and Alabama, the odds of respondents in South Carolina reporting long-acting reversible contraception use were significantly higher (24%) compared with respondents from Alabama. The odds of intrauterine device use increased significantly by 19%.

**Meaning:**

The South Carolina contraceptive access initiative may have increased use of long-acting reversible contraception methods (specifically intrauterine devices) among populations that have historically faced barriers in accessing these methods.

## Teaser

This cohort study of women of reproductive age in South Carolina examines changes in contraceptive method use during a contraceptive access initiative.

## Introduction

During the past several decades, multiple statewide contraceptive access initiatives have been implemented across the US. Although scale and scope may vary, these initiatives are primarily focused on increasing provision and use of a full range of contraceptive methods among people facing financial and clinical barriers to care.^1^ The South Carolina Choose Well contraceptive access initiative is the first and only initiative of its kind in the US Southeast. The need for a statewide contraceptive access initiative in South Carolina was informed by the high rate of unintended pregnancy in the state^2^ combined with lower rates of contraceptive use^3^ and large segments of the population facing barriers to care.^4^ The state’s Medicaid program was not expanded under the Patient Protection and Affordable Care Act, leaving many people with lower incomes without insurance coverage.^5^

The initiative launched in 2017 with a focus on promoting equitable access to contraception without coercion.^6^ Choose Well was designed as a 6-year initiative (2017-2022) with plans to sustain the initiative beyond its initial funding period. Originally designed to narrow contraceptive access gaps for the uninsured and underinsured, many individuals benefit from Choose Well activities, regardless of ability to pay.^6^ Choose Well focused on ensuring contraceptive access by offsetting the cost of contraceptives, developing infrastructure and workforce, capacity building and training, marketing and communications, and strategic learning and sustainability across various clinical sectors. Choose Well is estimated to have served more than 450 000 individuals and worked with approximately 121 unique partners across the state, including Federally Qualified Health Centers (FQHCs), Title X health department clinics, rural health clinics, delivering hospitals, and college health centers. Every county in the state was served by Choose Well activities, including a comprehensive marketing campaign. Central components of the initiative have been described elsewhere.^6,7^

Although safety-net clinics provide an important point of access for reproductive health care for people with lower incomes in the state, the range of services and contraceptive methods available vary widely among clinics.^8,9,10,11^ In 2016, fewer than 1 in 10 FQHCs in South Carolina offered the full range of contraceptive methods (intrauterine devices [IUDs], implants, pills, patch, ring, hormonal injection, diaphragm, and condoms), and IUDs were the least available method, with only 37% of the 19 Choose Well participating FQHCs surveyed offering any IUD on site.^9,10^ At midline of the formal implementation period (2019), significant increases in provision of the full range of contraceptive methods among Choose Well participating FQHCs in South Carolina were noted, with 85% of 48 Choose Well participating FQHCs offering IUDs on site.^9,10^ Choose Well implementation was also associated with significant increases in the use of IUD methods among South Carolina Medicaid beneficiaries at midline.^12^ These findings indicate that Choose Well succeeded in reducing barriers to contraceptive access for women with lower incomes during its initial 3 years of implementation.

Although these studies are encouraging and provide evidence in support of increased access to contraceptive methods, they are limited and up to this point have focused primarily on individuals enrolled in a single state Medicaid program. This study expands on previous research by examining changes in contraceptive method use, primarily IUDs and implants, between 2018 and 2021, among a population-based cohort of women of reproductive age in South Carolina compared with a peer state in the southeast (Alabama). South Carolina and Alabama have similar demographic characteristics and political environments. Although the South Carolina initiative is focused on expanding access to all contraceptive methods and ensuring person-centered contraceptive counseling, provision of IUDs and implants is subject to more financial and implementation barriers than other methods.^13,14^ The high upfront cost of devices in the absence of full insurance coverage and the need for specialized training, coupled with limitations to clinician scope of practice and other system-level regulations, create barriers in accessing these methods.^15,16,17,18,19,20^ Given the ongoing focus of Choose Well on removing barriers and ensuring access to contraception, the extent to which use of these methods has increased in South Carolina compared with a peer state remains a salient point of investigation.

## Methods

### Study Population and Data Source

Data were derived from the Statewide Survey of Women in Alabama and South Carolina, a probability-based sample of women of reproductive age (18-44 years), administered between October 1, 2017, and April 30, 2018. Women, hereafter referred to as participants, include those responding “female” when asked in the survey screener, “What is your sex?” Households were randomly selected from an address-based sampling frame. Survey methods and weighting procedures have been described elsewhere.^21,22^ Respondents from the initial cross-sectional sample were invited to complete 3 follow-up surveys occurring in 2019, 2020, and 2021, creating a longitudinal cohort. This study was reviewed by the East Tennessee State University Institutional Review Board and deemed non–human participant research because these data were collected by NORC at the University of Chicago and provided to the research team as a deidentified data set; therefore, no informed consent was needed. The Strengthening and Reporting of Observational Studies in Epidemiology (STROBE) reporting guidelines were followed in this study.

Cohort respondents were asked the same questions about contraception use and behaviors assessed during the initial survey. Responses from the initial cross-sectional survey were linked with responses from the follow-up surveys to create a longitudinal file for analysis. The initial cross-sectional survey yielded 4132 responses. Respondents who reported having a tubal ligation, being infertile, being currently pregnant, or trying to get pregnant were removed from the sample. Those reporting not wanting to be contacted for follow-up were also excluded, leaving 2262 participants. Of those eligible for follow-up, any respondent who reported being pregnant or trying to become pregnant during the follow-up period was removed. Of the remaining 2009 respondents, an additional 665 did not complete at least 2 of the 3 follow-up surveys (Figure). A total of 1344 of respondents remained and were included in the final study population (667 in Alabama and 677 in South Carolina), representing 59% of the initially eligible pool of participants (n = 2262).

**Figure.**
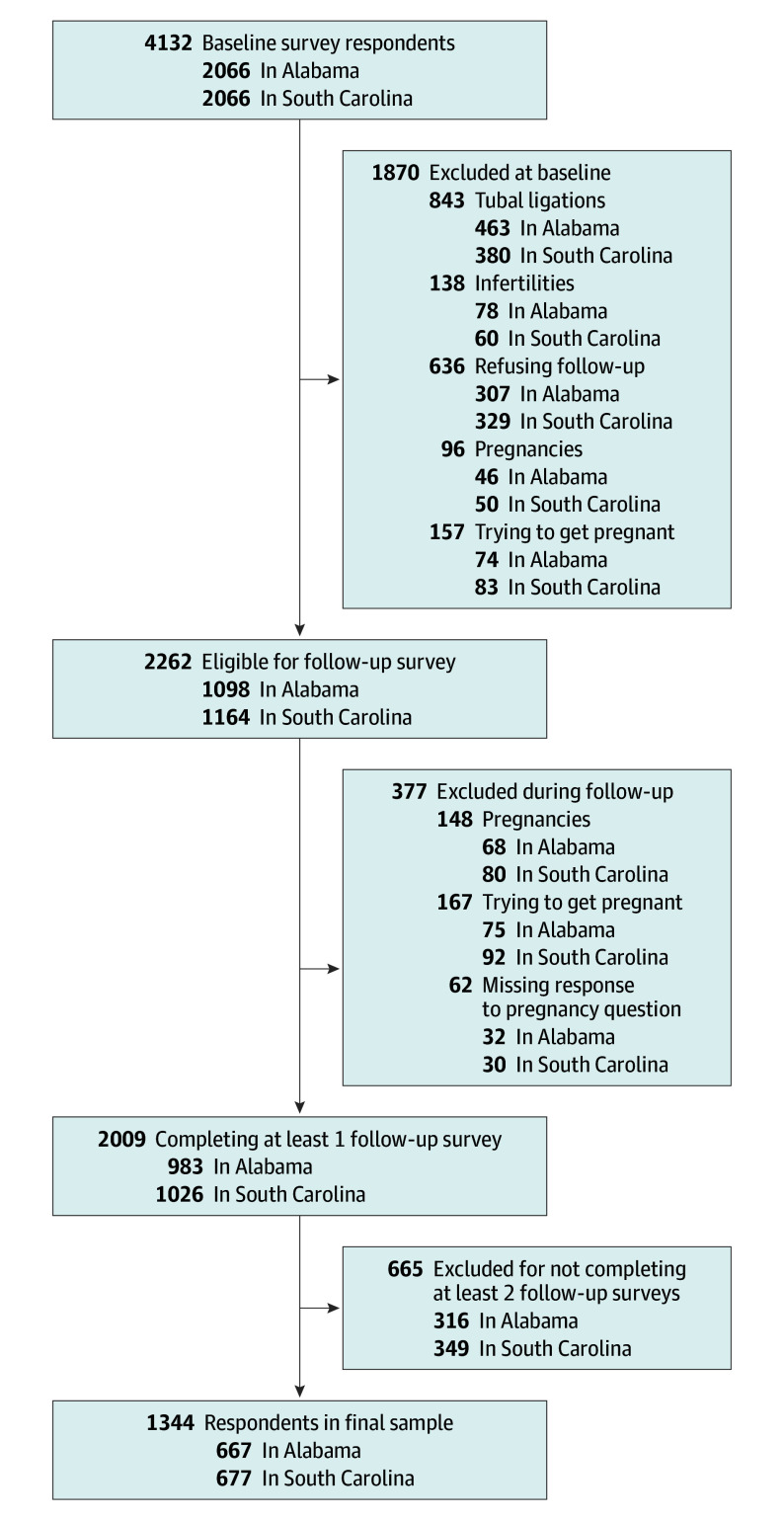
Participants in the Statewide Survey of Women Longitudinal Cohort Study (2018-2021) by Criteria and State

### Measures

Changes in contraception use were of primary interest. Those who responded no when asked, “Are you currently using any method or methods of birth control?” were categorized as nonusers. Respondents answering yes were subsequently asked, “What kind(s) of birth control method(s) are you currently using?” Given the cohort sample size by state, a categorical variable grouping individual methods was constructed. Implants and IUDs were categorized as long-acting reversible contraceptives (LARCs) and were also examined individually. Birth control pills, patches, hormonal injections, and vaginal rings were categorized as short-acting hormonal methods. Other methods (withdrawal, male condoms, natural family planning, and other female barrier methods) were also grouped as barrier or other methods analysis. When multiple methods were selected, the highest level of method effectiveness was retained.

The Andersen Behavioral Model of Health Services Utilization was used as the guiding framework for covariate selection of predisposing, enabling, and need factors that could potentially explain differences in the use of services. These covariates, which were self-reported through the initial survey, include age, race and ethnicity, marital status, educational attainment, insurance status, ability to afford birth control, contraceptive visit within the past 12 months, Person-Centered Contraceptive Counseling (PCCC) scale,^23,24^ and sexual activity with a male.

### Statistical Analysis

Characteristics of the study sample were described and bivariate differences by state at initial survey examined using the χ^2^ test of independence with a 2-sided significance level of *P* < .05. The study used a difference-in-differences method in which the year 2018 was treated as the initial year of the Choose Well Initiative and the years 2019 through 2021 were treated as the follow-up period. Differences in reported use or nonuse of any contraceptive method, LARC, IUD, implant, short-acting hormonal method, and other or barrier method were examined. To account for the longitudinal structure of the data and within-individual correlation from repeated measures of participants, generalized estimating equation models with binomial logit models and an exchangeable covariance matrix were used in the analysis. An interaction term for the year of the initiative and exposure state was used to derive a coefficient representing difference in changes in the use of contraceptive methods between states, comparing the initial year with the follow-up years. Coefficients were exponentiated and odds ratios (ORs) reflecting the differences in method use occurring in South Carolina compared with Alabama during the same period are presented. Adjusted models included age, race and ethnicity, educational attainment, marital status, insurance coverage, reported ability to afford birth control, having a contraceptive visit in the past year, and reported level of sexual activity. Only respondents with a reported contraceptive visit in the past year were asked to complete the PCCC scale and were not included in the adjusted models.

Additional analysis examined state differences in time-varying measures across follow-up survey periods, attrition among those eligible for follow-up but not included in the final sample, and attrition among those who started follow-up but did not complete at least 2 of the 3 surveys. These results are provided in eTables 1 to 3 in Supplement 1. All analyses were conducted from October 2023 to February 2024 using Stata software, version MP 15.0 (StataCorp LLC).

## Results

The longitudinal cohort included 1344 participants (mean [SD] age, 34 [7] years; 40 [3.4%] Hispanic or Latina, 236 [18.1%] non-Hispanic Black, 980 [75.3%] non-Hispanic White, and 46 [3.4%] non-Hispanic other, including American Indian or Alaska Native, Asian, Native Hawaiian or Pacific Islander, and multiple races) who completed the initial survey and at least 2 follow-up surveys (Table 1). A total of 667 (49.6%) of the study population resided in Alabama compared with 677 (50.4%) in South Carolina. No differences in the initial characteristics of the longitudinal cohort of participants were noted between the 2 states for age, race and ethnicity, insurance status, reported affordability of birth control, having a contraceptive visit in the past year, and sexual activity. Higher proportions of participants in South Carolina reported never being married compared with respondents in Alabama (279 [41.7%] vs 244 [37.3%]; *P* = .03). Higher proportions of participants in South Carolina (371 [56.4%]) reported having a bachelor’s degree or higher compared with respondents in Alabama (307 [47.5%]; *P* < .001). No significant between-state differences across follow-up periods were noted for time variant measures included in the analysis (eTable 1 in Supplement 1).

**Table 1.** Characteristics of Participants in the Statewide Survey of Women Longitudinal Cohort Study by State at Initial Survey (2018)

Characteristic	Participants, No. (%)			*P* value
	Total (N = 1344)	Alabama (n = 667)	South Carolina (n = 677)	
Time invariant variables				
Age group, y				
18-24	207 (15.4)	107 (16.0)	100 (14.8)	.40
25-29	217 (16.2)	112 (16.8)	105 (15.5)	
30-35	321 (23.9)	167 (25.0)	154 (22.8)	
36-39	270 (20.1)	121 (18.1)	149 (22.0)	
40-44	239 (24.5)	160 (24.0)	169 (25.0)	
Race and ethnicity				
Hispanic or Latina	40 (3.4)	18 (2.8)	22 (3.4)	.12
Non-Hispanic Black	236 (18.1)	133 (20.6)	103 (15.7)	
Non-Hispanic White	980 (75.3)	470 (72.9)	510 (77.6)	
Non-Hispanic other^a^	46 (3.4)	24 (3.7)	22 (3.4)	
Time variant variables				
Marital status				
Married	688 (52.0)	343 (52.4)	345 (51.6)	.03
Divorced, separated, or widowed	113 (8.5)	68 (10.4)	45 (6.7)	
Never married	523 (39.5)	244 (37.3)	279 (41.7)	
Educational attainment				
High school or equivalent	168 (12.9)	103 (15.9)	65 (9.9)	<.001
Some college	458 (35.1)	236 (36.5)	222 (33.7)	
Bachelor’s degree or higher	678 (56.4)	307 (47.5)	371 (56.4)	
Health insurance				
None	101 (7.8)	55 (8.5)	46 (7.1)	.34
Private	822 (63.6)	397 (61.6)	425 (65.5)	
Public	249 (19.3)	134 (20.8)	115 (17.7)	
Other	63 (9.7)	58 (9.0)	63 (9.75)	
Not able to afford birth control				
No	1225 (94.4)	626 (94.9)	629 (94.0)	.51
Yes	74 (5.6)	34 (5.2)	40 (6.0)	
Sexually active in past 3 mo				
Not active	378 (28.1)	198 (29.7)	180 (26.6)	.25
Active	966 (71.9)	469 (70.3)	497 (73.4)	
Contraceptive visit in past 12 mo				
No	546 (41.1)	275 (41.7)	271 (40.5)	.64
Yes	783 (58.9)	384 (58.3)	399 (59.6)	
Optimal contraceptive counseling^b^				
No	364 (44.4)	179 (44.6)	185 (44.3)	.91
Yes	455 (55.6)	222 (55.7)	233 (55.7)	

^a^ Non-Hispanic other includes American Indian or Alaska Native, Asian, Native Hawaiian or Pacific Islander, and multiple races.

^b^ Respondents are a subset representing those reporting a contraceptive visit in the past 12 months.

Noted in Table 2, reported LARC use increased by 3.5 percentage points in South Carolina (119 [17.6%] to 138 [21.1%]) compared with a 0.1–percentage point increase in Alabama (120 [18.0%] to 116 [18.1%]; *P* = .004). These findings persisted in the adjusted analysis with higher odds of reported LARC use at follow-up in South Carolina compared with Alabama (adjusted OR, 1.24; 95% CI, 1.06-1.44) (Table 2). A 3.4–percentage point increase in IUD use was noted in South Carolina (95 [14.0%] to 114 [17.4%]) compared with a 2.1–percentage point increase in Alabama (92 [13.8%] to 102 [15.9%]; *P* = .003). These associations persisted in the adjusted analysis, with a higher odds of reported IUD use among participants in South Carolina compared with Alabama at follow-up (adjusted OR, 1.19; 95% CI, 1.06-1.32) (Table 2). No differences in any method, short-acting hormonal methods, or barrier or other method use were noted in the bivariate or adjusted analysis (Table 2).

**Table 2.** Contraceptive Method Use at Initial Survey and Follow-Up Among Participants in the Statewide Survey of Women Longitudinal Cohort Study in Alabama and South Carolina (2018-2021)

Contraceptive method	Baseline survey (2018), No. (%)		Follow-up surveys (2019-2021), No. (%)		Odds ratio (95% CI)^a^		*P* value^c^
	Alabama (n = 667)	South Carolina (n = 677)	Alabama (n = 642)	South Carolina (n = 654)	Unadjusted	Adjusted^b^	
Any method^d^	448 (67.2)	462 (68.2)	421 (65.5)	431 (65.9)	0.99 (0.83-1.18)	1.04 (0.81-1.33)	.86
LARC	120 (18.0)	119 (17.6)	116 (18.1)	138 (21.1)	1.24 (1.07-1.43)	1.24 (1.06-1.44)	.004
IUD	92 (13.8)	95 (14.0)	102 (15.9)	114 (17.4)	1.16 (1.05-1.28)	1.19 (1.06-1.32)	.003
Implant	28 (4.2)	24 (3.5)	19 (3.0)	24 (3.7)	1.35 (0.92-1.99)	1.23 (0.76-1.98)	.12
Short-acting hormonal method^e^	375 (56.2)	376 (55.5)	381 (59.3)	380 (58.0)	1.08 (0.88-1.33)	1.09 (0.82-1.45)	.47
Barrier or other method^f^	98 (14.7)	105 (15.5)	90 (14.0)	87 (13.3)	0.98 (0.76-1.27)	1.04 (0.78-1.39)	.90

Abbreviations: IUD, intrauterine device; LARC, long-acting reversible contraception.

^a^ Odds of method use during the follow-up period in South Carolina relative to Alabama. Coefficients were derived from the interaction term between state exposure and year using unadjusted xtgee binomial logit models with an exchangeable covariance matrix.

^b^ Model adjusted for age, race and ethnicity, marital status, educational attainment, reported ability to afford birth control, contraceptive visit in the past 12 months, and reported sexual activity.

^c^ *P* value for state differences in method use between the initial survey and follow-up surveys. Values were derived from interaction term between state exposure and year using xtgee binomial logit models with an exchangeable covariance matrix.

^d^ Method counts do not sum to the total for any method use. Any method use was derived from a self-reported yes when asked, “Are you currently using any method or methods of birth control?” Participants may select no but subsequently check yes to individual methods listed in the survey. Method counts are based on selecting yes to an individual method not self-reported method use.

^e^ Excluding reported LARC use during same survey period.

^f^ Excluding reported LARC or short-acting hormonal method use during same survey.

State differences were examined in the characteristics of the initial cross-sectional sample eligible for inclusion in follow-up and those who were retained in the final sample. The final sample included greater representation of participants who were 30 years or older, White, more educated, and privately insured (eTable 2 in Supplement 1). However, the only significant differences between states was a lower odds of non-Hispanic Black participants being in the South Carolina sample compared with the findings in Alabama. Importantly, no state differences in baseline method use were observed between the 2 states. Similarly, no systematic state differences in attrition among those in the sample but lost to follow-up were noted (eTable 3 in Supplement 1).

## Discussion

This study examined changes in contraceptive use between 2018 and 2021 among a population-based cohort of women of reproductive age in South Carolina, where the Choose Well initiative was implemented, and a peer southeastern state. An increase in LARC use was noted in South Carolina that was not observed in another demographically similar state with no comparable contraceptive access initiative. Observed increases in LARC use in the South Carolina cohort were driven by increases in IUD use that were not observed among a similar cohort in Alabama. Importantly, there were no observed differences in time variant measures included in this study or known factors that could potentially explain differences in method use between the 2 states. These findings provide early evidence that the South Carolina initiative, implemented in a politically conservative state with restrictive reproductive health policies, may be associated with the increased use of methods with historical clinical and financial barriers to access.

A recent Choose Well evaluation study examining changes in contraception use patterns among women enrolled in the South Carolina Medicaid program between 2012 and 2020 found that LARC use increased from 8.5% during the preinitiative period to 10.9% during the initiative period, with significant increases in IUD use specifically.^12^ Another study found an increasing trend in IUD use among all Medicaid participants during the intervention period beyond what would be expected if pre–Choose Well trends had continued, which was particularly true for participants aged 20 to 25 years.^15^ That study examined changes in contraception use at the midpoint of the Choose Well initiative but with a broader cohort and different methods than the Medicaid study.

Another recent study examined the association of the South Carolina initiative with contraceptive provision at FQHCs at midline and concluded that the initiative’s efforts focused on removing cost barriers, capacity building, and clinician training had translated to increased provision of the full range of contraceptive methods at participating FQHCs in South Carolina.^9^ Taken together, these studies suggest that increased access to contraceptive methods, including IUDs, during the initial years of the South Carolina Choose Well initiative has translated to increased use among varying population groups. The extent to which increased provision and use of long-acting contraceptive methods holds over the full Choose Well implementation period remains to be seen.

Findings from previous contraceptive access initiatives in other regions of the US have noted similar trends, with increasing method provision and use after intervention support.^25,26,27,28^ These evaluation studies also reported changes in adolescent births, preterm birth rates, rates of unintended or mistimed pregnancy, and abortion with increased provision and use of contraceptive methods.^25,27,28,29^ The extent to which changes in method use observed in South Carolina translate to changes in reproductive health outcomes is currently under study.

Reproductive health equity is rooted in contraception choice and autonomy over reproductive health goals.^30,31^ Choose Well aimed to expand access to contraceptive options and supported the provision of IUDs and implants given the high costs of these methods and limited clinician training and supportive clinic policies.^32,33,34^ Ensuring all methods are available and affordable is an important component of reproductive choice and equitable access to care.^35^

Equally important is the provision of contraception without pressure and consistent with patient values and preferences. Choose Well provided trainings in PCCC to clinical partners across the state in support of its mission to promote access without judgment or coercion.^6,9,36^ Our study examined respondents’ assessment of receipt of person-centered contraceptive counseling among those who reported a contraceptive visit in the past year. Reported optimal counseling, as measured by the validated PCCC scale, increased across follow-up surveys compared with baseline, particularly in South Carolina. Furthermore, early findings from the longitudinal study of women, which recruits patients from Choose Well participating clinics across South Carolina and from matched nonparticipating clinics, also noted that a significantly greater percentage of patients recruited from Choose Well participating clinics completely agreed that their practitioner respected them as a person, took their preferences about birth control seriously, and let them say what mattered to them about their birth control method.^37^ These findings provide supporting evidence that observed increases in LARC method use during the South Carolina initiative were not associated with coercive counseling.

### Strengths and Limitations

This study has several strengths. A longitudinal cohort that includes participants from a broader population base was created, and no evidence of systematic state differences was present. These factors allowed for examining state differences in highly effective method use during a statewide contraceptive access initiative. Our finding that IUD use increased in the intervention state beyond what was observed in a peer state with no statewide access initiative is consistent with previously published research from the Choose Well evaluation.

This study also has some limitations. The study is focused on a longitudinal cohort of women originating from an initial, representative, population-based, cross-sectional survey. The characteristics, motivations, and experiences of women who completed follow-up surveys may be different than the initial population-based, cross-sectional survey of women. Analyses comparing participants from the original cross-sectional sample eligible for follow-up but not included in the longitudinal cohort found between-state differences in race and ethnicity. We also did not find any evidence of state differences in the use of LARC methods between the cohort and those eligible but not included; nor did we find any evidence of systematic state differences in attrition within the cohort. It is possible that preferential selection of IUD methods among White participants could explain some of the overall increases in IUD use observed in South Carolina compared with Alabama. However, nationally representative data from the same period indicate that overall LARC use rates between non-Hispanic White and non-Hispanic Black women were nearly equal.^38^ Furthermore, previous research examining contraceptive method use between South Carolina and Alabama from the initial cross-sectional sample noted that LARC use was higher among non-Hispanic Black respondents than among non-Hispanic White participants in both South Carolina and Alabama.^22^ The longitudinal cohort may not be representative of the overall population in each state. However, this study is focused on examining differences in method use between states during a contraceptive access initiative among a cohort of participants and is not intended to produce population-level estimates of method use rates. We did not find evidence of systematic state differences that could explain observed differences in method use in South Carolina compared with Alabama. These findings suggest that observed increases in IUD use in South Carolina that were not observed in Alabama were not the result of systematic state differences or preferential selection among White participants, although contraceptive preferences were not included in the analysis. It is also possible that nonuse might have been misclassified among some respondents who did not perceive methods such as condoms or withdrawal as contraceptive methods.

## Conclusions

Our study may provide evidence in support of the South Carolina Choose Well initiative on contraceptive method use among a longitudinal, population-based cohort of women of reproductive age in the state. The South Carolina Choose Well initiative is the first intervention of its kind in the US Southeast, a region with restrictive sexual and reproductive health policies. Our findings may contribute to an increasing evidence base in support of statewide contraceptive access initiatives and their role in promoting access to reproductive health services and advancing health equity among diverse population groups.

## Notes

Supplement 1. **eTable 1.** Time Variant Measures at Initial Survey and Follow Up among Participants in the Statewide Survey of Women Longitudinal Cohort in Alabama and South Carolina (2018-2021) **eTable 2.** Characteristics of Participants Eligible for Follow Up Not Included in Final Sample in Alabama and South Carolina (2018-2021) **eTable 3.** Characteristics of Participants Lost to Follow Up After Completing At Least One Follow Up Survey in Alabama and South Carolina (2018-2021)

Supplement 2.
Data Sharing Statement
