# Supplementary material for: Contraceptive Method Use Among a Population-Based Cohort During the South Carolina Choose Well Initiative
Source: JAMA Netw Open. 2024 Apr 24;7(4):e248262. doi: 10.1001/jamanetworkopen.2024.8262 (PMC11043899; doi:10.1001/jamanetworkopen.2024.8262)
Supplement: Supplement 1. — eTable 1. Time Variant Measures at Initial Survey and Follow Up among Participants in the Statewide Survey of Women Longitudinal Cohort in Alabama and South Carolina (2018-2021) eTable 2. Characteristics of Participants Eligible for Follow Up Not Included in Final Sample in Alabama and South Carolina (2018-2021) eTable 3. Characteristics of Participants Lost to Follow Up After Completing At Least One Follow Up Survey in Alabama and South Carolina (2018-2021) [file jamanetwopen-e248262-s001.pdf]

Supplementary Online Content

Hale N, Lee J, de Jong J, Smith MG, Khoury AJ. Contraceptive method use among a population-based cohort during the South Carolina Choose Well initiative. *JAMA Netw Open*. 2024;7(4):e248262. doi:10.1001/jamanetworkopen.2024.8262

eTable 1. Time Variant Measures at Initial Survey and Follow Up among Participants in the Statewide Survey of Women Longitudinal Cohort in Alabama and South Carolina (2018-2021)

eTable 2. Characteristics of Participants Eligible for Follow Up Not Included in Final Sample in Alabama and South Carolina (2018-2021)

eTable 3. Characteristics of Participants Lost to Follow Up After Completing At Least One Follow Up Survey in Alabama and South Carolina (2018-2021)

This supplementary material has been provided by the authors to give readers additional information about their work.

© 2024 Hale N et al. *JAMA Network Open.*

eTable1: Time Variant Measures at Initial Survey and Follow Up among Participants in the Statewide Survey of Women Longitudinal Cohort in Alabama and South Carolina (2018-2021)

	Participant No. (%)[a]								*P* Value[b]
	Baseline Survey (2018)		Follow-Up 1 (2019)		Follow-Up 2 (2020)		Follow-Up 3 (2021)		
	Alabama	South Carolina	Alabama	South Carolina	Alabama	South Carolina	Alabama	South Carolina	
	667 (49.6)	677 (50.4)	612 (50.0)	613 (50.0)	632 (49.7)	639 (50.3)	620 (49.6)	631 (50.4)	
Time Variant Variables									
Marital status									
Married	343 (52.4)	345 (51.6)	316 (52.5)	340 (56.1)	328 (52.3)	331 (52.2)	341 (55.5)	331 (52.7)	Ref.
Divorced/separated/ widowed	68 (10.4)	45 (6.7)	71 (11.8)	43 (7.1)	80 (12.8)	51 (8.0)	74 (12.1)	60 (9.6)	0.96
Never married	244 (37.3)	279 (41.7)	215 (35.7)	223 (36.8)	219 (34.9)	252 (39.8)	199 (32.4)	237 (37.7)	0.58
Educational attainment									
High school or equivalent	103 (15.9)	65 (9.9)	98 (16.2)	58 (9.5)	103 (16.4)	60 (9.5)	96 (15.6)	54 (8.6)	Ref.
Some college	236 (36.5)	222 (33.7)	209 (34.5)	199 (32.7)	210 (33.4)	211 (33.2)	201 (32.6)	206 (32.7)	0.71
Bachelor's degree or higher	307 (47.5)	371 (56.4)	299 (49.3)	352 (57.8)	316 (50.2)	364 (57.3)	320 (51.9)	370 (58.7)	0.83
Health Insurance									
No	55 (8.5)	46 (7.1)	50 (8.4)	47 (7.9)	49 (7.9)	55 (8.8)	40 (6.2)	41 (6.7)	0.41
Private	397 (61.6)	425 (65.5)	356 (59.8)	367 (61.4)	351 (56.5)	369 (59.1)	356 (58.9)	378 (61.3)	Ref.
Public	134 (20.8)	115 (17.7)	100 (16.8)	100 (16.7)	108 (17.4)	97 (15.5)	106 (17.6)	104 (16.9)	0.47
Other	58 (9.0)	63 (9.75)	89 (15.0)	84 (14.1)	113 (18.2)	103 (16.5)	102 (16.9)	94 (15.2)	0.94

© 2024 Hale N et al. *JAMA Network Open.*

Not Able to Afford Birth Control									
No	626 (94.9)	629 (94.0)	574 (95.2)	566 (94.5)	593 (95.3)	601 (95.3)	595 (96.4)	600 (96.3)	Ref.
Yes	34 (5.2)	40 (6.0)	29 (4.8)	33 (5.5)	29 (4.7)	30 (4.8)	22 (3.6)	23 (3.7)	0.76
Sexually Active in past 3 months									
Not active	159 (25.3)	145 (22.6)	145 (25.1)	132 (22.2)	143 (23.8)	144 (23.4)	149 (25.3)	145 (24.0)	Ref.
Active	469 (74.7)	497 (77.4)	433 (74.9)	462 (77.8)	459 (76.3)	471 (76.6)	441 (74.8)	460 (76.0)	0.99
Contraceptive Visit in Past 12 months[b]									
No	275 (41.7)	271 (40.5)	27 (45.1)	284 (46.6)	302 (47.9)	321 (50.7)	297 (48.1)	342 (54.6)	Ref.
Yes	384 (58.5)	399 (59.6)	334 (54.9)	325 (53.4)	329 (52.1)	312 (49.3)	320 (51.9)	284 (45.4)	0.61
Optimal Counseling [c]									
No	179 (44.6)	185 (44.3)	145 (42.4)	142 (42.6)	133 (39.2)	116 (35.6)	122 (37.2)	93 (31.3)	Ref.
Yes	222 (55.7)	233 (55.7)	197 (57.6)	191 (57.4)	206 (60.8)	210 (64.4)	206 (62.8)	204 (68.7)	0.51

[a] Unweighted n's and Percentages

[b] P value for state differences in measure at initial survey and across follow-up surveys. Derived from interaction term between state and year for each measure.

[c] Respondents are a subset representing those reporting a contraceptive visit in the past 12 months.

© 2024 Hale N et al. *JAMA Network Open.*

eTable2: Characteristics of Participants Eligible for Follow Up Not Included in Final Sample in Alabama and South Carolina (2018-2021)

	No.(%)		*P* Value [a]	No.(%)		*P* Value [a]	*P* Value [b]
	Alabama			South Carolina			
	In Sample	Not In Sample		In Sample	Not In Sample		
	n=667 (49.6)	n=316 (47.5)		n=677 (50.4)	n=349 (52.5)		
**Time Invariant Variables**							
Age							
18-24	107 (16.0)	121 (28.1)	<0.001	100 (14.8)	107 (22.0)	<0.001	Ref.
25-29	112 (16.8)	86 (20.0)		105 (15.5)	92 (18.9)		0.50
30-35	167 (25.0)	85 (19.7)		154 (22.8)	122 (25.1)		0.06
36-39	121 (18.1)	62 (14.4)		149 (22.0)	81 (16.6)		0.67
40-44	160 (24.0)	77 (17.9)		169 (25.0)	85 (17.5)		0.72
Race and Ethnicity							
Hispanic/Latina	18 (2.8)	15 (3.6)	<0.001	22 (3.4)	17 (3.6)	<0.001	0.89
Non-Hispanic Black	133 (20.6)	99 (23.5)		103 (15.7)	125 (26.7)		0.02
Non-Hispanic White	470 (72.9)	281 (66.8)		510 (77.6)	302 (64.5)		Ref.
Non-Hispanic Other[b]	24 (3.7)	26 (6.2)		22 (3.4)	24 (5.1)		0.97
**Time Variant Variables**							
Marital status							
Married	343 (52.4)	203 (48.0)	0.33	345 (51.6)	231 (48.6)	0.44	Ref.
Divorced/separated/widowed	68 (10.4)	44 (10.4)		45 (6.7)	40 (8.4)		0.54
Never married	244 (37.3)	176 (41.6)		279 (41.7)	204 (43.0)		0.55
Educational attainment							
High school or equivalent	108 (16.4)	90 (21.0)	0.006	67 (9.9)	87 (18.1)	<0.001	Ref
Some college	244 (37.0)	180 (42.0)		233 (34.5)	168 (34.9)		0.07
Bachelor's degree or higher	308 (46.7)	159 (37.1)		375 (55.6)	227 (47.1)		0.26
Health Insurance							

© 2024 Hale N et al. *JAMA Network Open.*

None	55 (8.5)	46 (11.3)	0.07	46 (7.1)	31 (6.8)	0.02	0.27
Private	397 (61.6)	219 (53.8)		425 (65.5)	271 (59.2)		Ref.
Public	134 (20.8)	103 (25.3)		115 (17.7)	117 (25.6)		0.53
Other	58 (9.0)	39 (9.6)		63 (9.75)	39 (8.5		0.46
Not Able to Afford Birth Control							
No	626 (94.9)	395 (93.2)	0.25	629 (94.0)	455 (94.8)	0.57	Ref.
Yes	34 (5.2)	29 (6.8)		40 (6.0)	25 (5.2)		0.23
Sexually active in past 3 months							
Not active	198 (29.7))	115 (26.8)	0.28	180 (26.6)	122 (25.1)	0.55	Ref.
Active	469 (70.3)	316 (73.3)		497 (73.4)	365 (75.0)		0.73
Contraceptive visit in past 12 months							
No	275 (41.7)	175 (41.6)	0.96	271 (40.5)	197 (41.2)	0.80	Ref.
Yes	384 (58.3)	246 (58.4)		399 (59.6)	281 (58.8)		0.82
Optimal Contraceptive Counseling [c]							
No	179 (44.6)	115 (42.9)	0.66	185 (44.3)	140 (46.5)	0.55	Ref.
Yes	222 (55.7)	153 (57.1)		233 (55.7)	161 (53.4)		0.46
Method Use							
Any Method Use	448 (67.2)	264 (61.3)	0.12	462 (68.2)	304 (62.4)	0.12	0.98
LARC	120 (18.0)	59 (13.7)	0.06	119 (17.6)	91 (18.7)	0.62	0.08
IUD	92 (13.8)	41 (9.5)	0.03	95 (14.0)	65 (13.4)	0.74	0.17
Implant	30 (4.5)	18 (4.2)	0.80	25 (3.7)	27 (5.5)	0.13	0.23
SAC	211 (38.6)	150 (40.3)	0.59	206 (36.9)	141 (35.6)	0.68	0.50
Barrier/Other	99 (29.5)	70 (31.5)	0.60	107 (30.4)	86 (33.7)	0.39	0.83

[a] Chi-square test for independence examining within state differences in the study sample and those lost to follow up.
[b] P value for state differences in lost to follow up within the initial cohort relative to the final sample. Values derived from a logistic regression model with lost to follow up as the primary dependent variable and the interaction term state of exposure and measures of interest as the primary independent variable.

© 2024 Hale N et al. *JAMA Network Open.*

eTable3: Characteristics of Participants Lost to Follow Up After Completing At Least One Follow Up Survey in Alabama and South Carolina (2018-2021)

	No.(%)		*P* Value [a]	No.(%)		*P* Value [a]	*P* Value [b]
	Alabama			South Carolina			
	Sample	Lost to Follow Up		Sample	Lost to Follow Up		
	n=667 (49.6)	n=316 (47.5)		n=677 (50.4)	n=349 (52.5)		
**Time Invariant Variables**							
Age							
18-24	107 (16.0)	85 (26.9)	<0.001	100 (14.8)	84 (24.7)	0.005	Ref.
25-29	112 (16.8)	54 (17.1)		105 (15.5)	56 (16.1)		0.89
30-35	167 (25.0)	50 (15.8)		154 (22.8)	63 (18.1)		0.40
36-39	121 (18.1)	55 (17.4)		149 (22.0)	67 (19.2)		0.83
40-44	160 (24.0)	72 (22.8)		169 (25.0)	79 (22.6)		0.95
Race and Ethnicity							
Hispanic/Latina	18 (2.8)	12 (3.9)	0.15	22 (3.4)	10 (3.0)	<0.001	0.46
Non-Hispanic Black	133 (20.6)	78 (25.3)		103 (15.7)	87 (26.3)		0.15
Non-Hispanic White	470 (72.9)	196 (63.6)		510 (77.6)	218 (65.9)		Ref.
Non-Hispanic Other	24 (3.7)	22 (7.1)		22 (3.4)	16 (4.8)		0.58
**Time Variant Variables**							
Marital status							
Married	343 (52.4)	138 (44.8)	0.09	345 (51.6)	154 (45.4)	0.14	Ref.
Divorced/separated/widowed	68 (10.4)	38 (12.3)		45 (6.7)	30 (8.9)		0.83
Never married	244 (37.3)	132 (42.9)		279 (41.7)	155 (45.7)		0.70
Educational attainment							
High school or equivalent	103 (15.9)	70 (22.3)	0.001	65 (9.9)	70 (20.4)	<0.001	Ref
Some college	236 (36.5)	136 (43.3)		222 (33.7)	125 (36.3)		0.06
Bachelor's degree or higher	307 (47.5)	108 (32.2)		371 (56.4)	149 (33.8)		0.20

© 2024 Hale N et al. *JAMA Network Open.*

Health Insurance							
None	55 (8.5)	34 (11.6)	0.09	46 (7.1)	24 (7.3)	0.02	0.41
Private	397 (61.6)	157 (53.4)		425 (65.5)	191 (58.4)		Ref.
Public	134 (20.8)	76 (25.8)		115 (17.7)	86 (26.3)		0.54
Other	58 (9.0)	27 (9.2)		63 (9.75)	26 (8.0)		0.48
Not Able to Afford Birth Control							
No	626 (94.9)	288 (92.9)	0.23	629 (94.0)	325 (94.8)	0.64	Ref.
Yes	34 (5.2)	22 (7.1)		40 (6.0)	18 (5.3		0.24
Sexually active in past 3 months							
Not active	198 (29.7))	101 (32.0)	0.47	180 (26.6)	105 (30.1)	0.24	Ref.
Active	469 (70.3)	215 (68.0)		497 (73.4)	244 (69.9)		0.75
Contraceptive visit in past 12 months							
No	275 (41.7)	148 (48.2)	0.06	271 (40.5)	157 (45.9)	0.10	Ref.
Yes	384 (58.3)	159 (51.8)		399 (59.6)	185 (54.1)		0.84
Optimal Contraceptive Counseling [c]							
No	179 (44.6)	69 (38.6)	0.17	185 (44.3)	95 (46.8)	0.55	Ref.
Yes	222 (55.7)	110 (61.5)		233 (55.7)	108 (53.2)		0.16

[a] Chi-square test for independence examining within state differences in the study sample and those lost to follow up.
[b] P value for state differences in lost to follow up within the initial cohort relative to the final sample. Values derived from a logistic regression model with lost to follow up as the primary dependent variable and the interaction term state of exposure and measures of interest as the primary independent variable.

© 2024 Hale N et al. *JAMA Network Open.*
