# Supplementary material for: Contraceptive Method Use Among a Population-Based Cohort During the South Carolina Choose Well Initiative
Source: JAMA Netw Open. 2024 Apr 24;7(4):e248262. doi: 10.1001/jamanetworkopen.2024.8262 (PMC11043899; doi:10.1001/jamanetworkopen.2024.8262)
Supplement: Supplement 2. — Data Sharing Statement [file jamanetwopen-e248262-s002.pdf]

Data Sharing Statement

Hale. Contraceptive Method Use Among a Population-Based Cohort During the South Carolina Choose Well Initiative. *JAMA Netw Open*. Published April 24, 2024. doi:10.1001/jamanetworkopen.2024.8262

Data
Data available: No

Additional Information
Explanation for why data not available: The research team is not at liberty to distribute the data per the Data Use Agreement with NORC the custodian of these survey data. Researchers may request these data directly from NORC at https://www.norc.org/research/projects/surveys-of-women.html
